# Gastric endoscopic submucosal dissection assisted by intralesional cross-traction using silicone bands

**DOI:** 10.1055/a-1982-3875

**Published:** 2022-12-13

**Authors:** Yusuke Suzuki, Mitsuru Esaki, Taisuke Inada, Yosuke Minoda, Haruei Ogino, Eikichi Ihara, Yoshihiro Ogawa

**Affiliations:** 1 Department of Medicine and Bioregulatory Science, Graduate School of Medical Sciences, Kyushu University, Fukuoka, Fukuoka, Japan; 2 Department of Gastroenterology and Metabolism, Graduate School of Medical Sciences, Kyushu University, Fukuoka, Fukuoka, Japan

Several traction techniques for endoscopic submucosal dissection (ESD) of gastric neoplasms have been reported to be useful 1 2 3 4 5 . We have previously reported an intralesional traction technique using a single traction band 5 . Since this creates a traction force within the lesion, theoretically, it could be applied to any gastric lesions. However, we do encounter cases in a few regions of the stomach where the traction force is insufficient. Here, we have proposed an intralesional cross-traction technique using dual traction bands ( Fig. 1 ), which might overcome the weaknesses of the previous method ( Video 1 ).

**Fig. 1:**
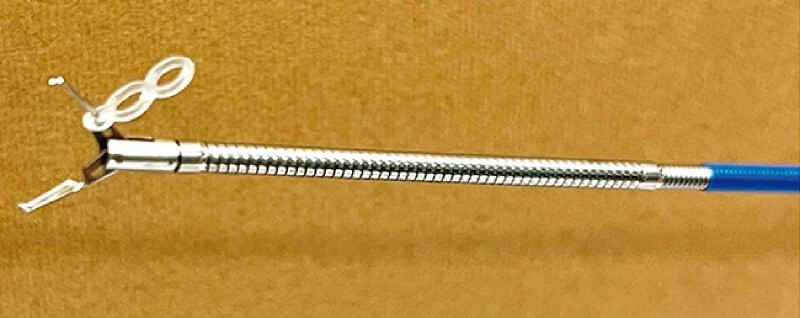
Endoscopic device for applying intralesional cross-traction: a clip with a silicone band at its base.

**Video 1** Intralesional cross-traction using silicone bands to assist gastric endoscopic submucosal dissection.


A 77-year-old man with a 20-mm gastric neoplasm in the lower stomach was referred to our hospital. ESD using the intralesional cross-traction method was applied. After the circumferential mucosal incision ( Fig. 2 a ), the first clip with a silicon band was placed on the proximal mucosal flap of the lesion and the second clip with a silicon band was placed at nearly one-fourth of the circumference away from the first clip ( Fig. 2 b ). The third clip, without a silicon band, was hooked through the silicon band of the first clip ( Fig. 2 c ) and placed on the mucosal flap opposite ( Fig. 2 d ). This was repeated with the fourth and the second clip ( Fig. 2 e ), in such a way that the bands crossed each other at the center of the lesion ( Fig. 2 f ). This resulted in the elevation of a large area of submucosal layer toward the center by cross-traction, providing a clear view for submucosal dissection that was conducted safely and efficiently The lesion was sufficiently elevated until the dissection was completed, and en bloc resection was achieved without complications.

**Fig. 2:**
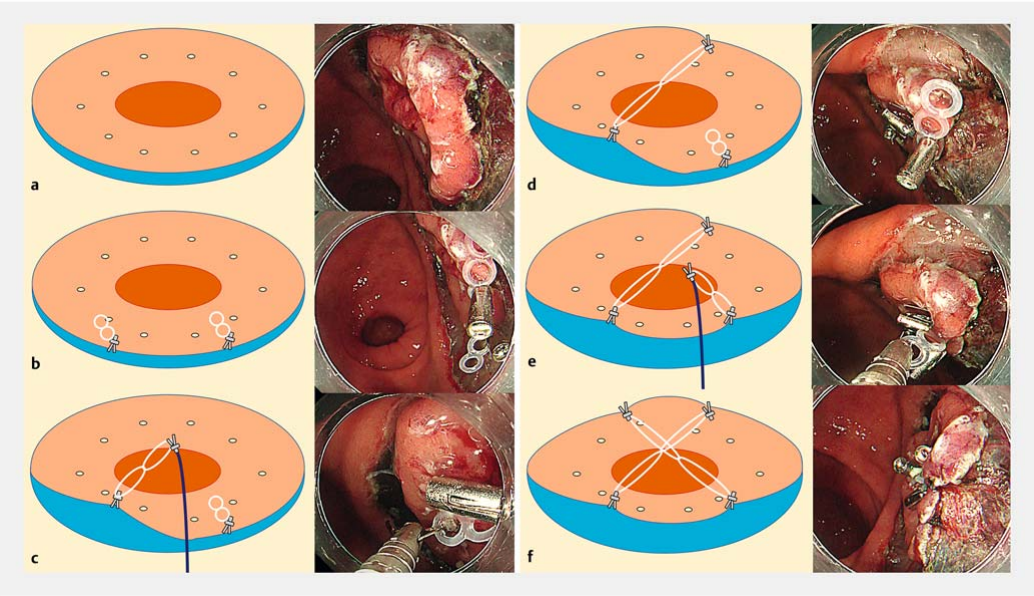
Schemata and representative endoscopic images showing each step followed when applying intralesional cross-traction. **a** A circumferential mucosal incision is performed around the marking dots. **b** The first clip with a silicon band is placed on the proximal mucosal flap of the lesion. The second clip with a silicon band is placed nearly one-fourth of the circumference away from the first clip. **c** The silicone band of the first clip is hooked with the third clip. **d** The third clip is placed on the mucosal flap at the opposite side from the first clip. **e** The silicon band of the second clip is hooked with the fourth clip. **f** The fourth clip is then placed on the mucosal flap at the opposite side from the second clip, thus crossing the bands at the center of the lesion.

The intralesional cross-traction method, which provides traction force over a large area and bundles the submucosal layer toward the center of the lesion, can be applied to any gastric lesion. This is one of the best assistive methods for gastric ESD.

Endoscopy_UCTN_Code_TTT_1AQ_2AD

## Notes

Endoscopy E-Videos

https://eref.thieme.de/e-videos
*Endoscopy E-Videos* is an open access online section, reporting on interesting cases and new techniques in gastroenterological endoscopy. All papers include a high quality video and all contributions are freely accessible online. Processing charges apply (currently EUR 375), discounts and wavers acc. to HINARI are available. This section has its own submission website at https://mc.manuscriptcentral.com/e-videos
